# Epigenome-wide association study identifies DNA methylation markers for asthma remission in whole blood and nasal epithelium

**DOI:** 10.1186/s13601-020-00365-4

**Published:** 2020-12-11

**Authors:** Cancan Qi, Judith M. Vonk, Diana A. van der Plaat, Maartje A. E. Nieuwenhuis, F. Nicole Dijk, BIOS Consortium, Dylan Aïssi, Valérie Siroux, H. Marike Boezen, Cheng-jian Xu, Gerard H. Koppelman

**Affiliations:** 1 Department of Pediatric Pulmonology and Pediatric Allergy, Beatrix Children’s Hospital, University Medical Center Groningen, University of Groningen, PO Box 30.001, 9700 RB Groningen, the Netherlands; 2 GRIAC Research Institute, University Medical Center Groningen, University of Groningen, Groningen, The Netherlands; 3 Department of Epidemiology, University Medical Center Groningen, University of Groningen, Groningen, The Netherlands; 4 University Grenoble Alpes, Inserm, CNRS, Team of environmental epidemiology applied to Reproduction and Respiratory health, IAB (Institute for Advanced Biosciences), 38000 Grenoble, France; 5 Research Group of Bioinformatics and Computational Genomics, CiiM, Centre for individualized infection medicine, A Joint Venture Between Hannover Medical School and the Helmholtz Centre for Infection Research, Hannover, Germany; 6 Department of Gastroenterology, Hepatology and Endocrinology, TWINCORE, Centre for Experimental and Clinical Infection Research, A Joint Venture Between the Hannover Medical School and the Helmholtz Centre for Infection Research, Hannover, Germany; 7 Department of Internal Medicine, Radboud University Medical Center, Nijmegen, the Netherlands

**Keywords:** Asthma remission, DNA methylation, Whole blood, Nasal brushes

## Abstract

**Background:**

Asthma is a chronic respiratory disease which is not curable, yet some patients experience spontaneous remission. We hypothesized that epigenetic mechanisms may be involved in asthma remission.

**Methods:**

Clinical remission (ClinR) was defined as the absence of asthma symptoms and medication for at least 12 months, and complete remission (ComR) was defined as ClinR with normal lung function and absence of airway hyperresponsiveness. We analyzed differential DNA methylation of ClinR and ComR comparing to persistent asthma (PersA) in whole blood samples (n = 72) and nasal brushing samples (n = 97) in a longitudinal cohort of well characterized asthma patients. Significant findings of whole blood DNA methylation were tested for replication in two independent cohorts, Lifelines and Epidemiological study on the Genetics and Environment of Asthma (EGEA).

**Results:**

We identified differentially methylated CpG sites associated with ClinR (7 CpG sites) and ComR (129 CpG sites) in whole blood. One CpG (cg13378519, Chr1) associated with ClinR was replicated and annotated to *PEX11* (Peroxisomal Biogenesis Factor 11 Beta). The whole blood DNA methylation levels of this CpG were also different between ClinR and healthy subjects. One ComR-associated CpG (cg24788483, Chr10) that annotated to *TCF7L2* (Transcription Factor 7 Like 2) was replicated and associated with expression of *TCF7L2* gene. One out of seven ClinR-associated CpG sites and 8 out of 129 ComR-associated CpG sites identified from whole blood samples showed nominal significance (P < 0.05) and the same direction of effect in nasal brushes.

**Conclusion:**

We identified DNA methylation markers possibly associated with clinical and complete asthma remission in nasal brushes and whole blood, and two CpG sites identified from whole blood can be replicated in independent cohorts and may play a role in peroxisome proliferation and Wnt signaling pathway.

## Introduction

Asthma is a chronic airway disorder that affects more than 300 million people around the world. Asthma cannot be completely cured with the available treatment up to now. However, some asthma patients may grow out of this disease, which is called asthma remission.

Asthma remission is more common in patients with childhood onset asthma, and the proportions of remission differ in different age groups at follow-up (33–53% in adolescence, 6–33% in young adulthood and 11–52% in adulthood) [1, 2]. There are two types of asthma remission, one is defined by absence of asthma symptoms and medication for at least one year, which is called “clinical remission” (ClinR). Some ClinR subjects may still have airway hyperresponsiveness (AHR) or a low lung function. Therefore, “complete remission” (ComR) was put forward [3]. In addition to criteria of ClinR, ComR has to meet additional criteria of normal lung function and absence of AHR. This is a more rare phenomenon, that takes place in 5–22% of asthmatics [2].

Asthma remission is associated with both genetic and environmental factors. Environmental factors including breast feeding and having pets in childhood were reported to be positively associated with asthma remission [4]. A recent genome-wide association study (GWAS) identified one SNP (single nucleotide polymorphism) that was associated with ClinR and three SNPs with ComR. One of these SNPs was also associated with expression of known asthma genes including *ILRL1* and *IL13* in lung tissue [5].

Epigenetic mechanisms such as DNA methylation may help to build a link between genetic factors and the environment. DNA methylation can be regulated by SNPs, and can reflect environmental exposures, ageing, cell type constitution and activation [6]. DNA methylation refers to the addition of a methyl-group to cytosine, which usually happens when a cytosine is located next to a guanine in the 5′ to 3′ direction (CpG site), and this may relate to the regulation of gene expression [7]. Epigenome-wide association studies (EWAS) have provided insights into the development of asthma and its remission. CpG sites associated with asthma in both blood and nasal epithelial cells have been identified, such as CpG sites located in *DICER1, STX3,* and *LIPIN1* in blood cells, and *CDHR3, FBXL7* and *NTRK1* in nasal epithelial cells [8, 9]. Vermeulen et al*.* [10] identified 4 CpG sites and 42 regions that were differentially methylated between remission and persistent asthma (PersA) in bronchial biopsies, and top CpG sites were annotated to genes including *ACKR2* and *DGKQ* by gene expression.

Although this latter paper provided a proof of concept of the relation between asthma remission and DNA methylation, bronchial biopsies are not easy to obtain for further studies. DNA methylation in whole blood and nasal epithelium is a good proxy for bronchial epithelium and can therefore also help to understand the mechanism of asthma remission. Here, we hypothesize that epigenetic mechanisms may be involved in asthma remission, reflected in different DNA methylation patterns in whole blood and nasal epithelium. To test this hypothesis, we performed an EWAS of whole blood and nasal DNA in subjects with PersA, ClinR and ComR. We investigated a longitudinal cohort in which asthma was initially carefully defined and the remission status was assessed during follow-up (median 39 years). We subsequently replicated the whole blood DNA results in two independent cohorts, and also verified the top results from whole blood DNA in cells obtained by nasal brushing.

## Methods

A full description of methods is provided in the online supplement.

### Study populations

Subjects included in this study were from long-term follow-up studies in the University Medical Center Groningen (UMCG). This study consists of two sources of subjects, (1) subjects from the third visit (2013–2014) of a longitudinal study described previously by Carpaij et al [4], and (2) additional asthma remission subjects that were followed up from previous genetic studies [11] and were re-invited in 2013–2014 to take nasal brushes and blood samples, during which the remission status was assessed again. The participants included in this cohort were different from those of the cohort described by Vermeulen et al. [10] from our research institute. All participants were diagnosed with asthma at baseline with a doctor diagnosis of asthma and AHR. Then, subjects had at least one follow-up medical examination during adulthood in which their asthma status was evaluated by questionnaires and in most subjects additionally with spirometry and an AHR test [5]. The medical ethical board of the UMCG approved the studies and all participants gave written informed consent.

We replicated findings in whole blood in two independent cohorts: the Lifelines population-based cohort in The Netherlands (where we could replicate our results on ClinR using Illumina 450 K array) and the Epidemiological study on the Genetics and Environment of Asthma (EGEA) cohort in France, a case control and family study on asthma, where we could replicate our results on ClinR and ComR with methylC-capture sequencing [12]. No cohort was available to replicate our nasal findings. Extensive information on these cohorts is described in the Additional file 1.

### Phenotype definition

The presence of PersA, ClinR and ComR was determined at the most recent visit. ClinR was defined according to the following criteria: (1) No use of any asthma medication, and (2) no symptoms (asthma attacks and/ or wheezing) in the past year. ComR was defined as ClinR combined with (3) no AHR (PC_20_ (provocative concentration causing a 20% fall in FEV_1_ (forced expiratory volume in 1 s)) methacholine ⩽ 39.3 mg mL^−1^)), and (4) FEV_1_% predicted pre-bronchodilator > 80%. PersA was defined as the presence of asthma symptom and/or the use of asthma medication. Detailed phenotype definitions of the replication cohorts are described in the Additional file 1.

### DNA methylation measurements and statistical analyses

DNA was extracted from 72 whole blood and 103 nasal brushing samples taken at the most recent visit. Genome-wide DNA methylation was determined using Illumina Infinium HumanMethylation450 BeadChips. After quality control, 72 whole blood samples and 97 nasal epithelium samples (of in total 103 unique subjects) with 436,824 CpG sites remained for following steps.

We used robust linear regression to determine the differential methylation between persistent asthma and asthma remission: (1) PersA versus ClinR, and (2) PersA versus ComR, in whole blood and nasal brushing samples, with adjustment for covariates that are known to affect DNA methylation (age, sex, smoking status, pack years, and batch). For whole blood samples, we performed adjustment on the percentage of monocytes, B cells, NK cells, CD4 + T cells, CD8 + T cells, and granulocytes, which were predicted by the Houseman [13] algorithm using minfi [14] package. For nasal brushing samples, we applied the R package sva [15] to estimate significant surrogate variables (SVs), representing unknown latent factors that capture heterogeneity in data, such as cell type composition. One SV for each analysis (ClinR and ComR) was generated and added to the model respectively. Differentially methylated regions (DMR) were identified using comb-p v0.48 [16] and DMRcate [17].

### Replication and meta-analyses

Genome-wide significant CpG sites that passed Bonferroni correction (P < 1.14 × 10^–7^, which is 0.05 / 436,824) in whole blood DNA were selected for replication in two independent cohorts, Lifelines and EGEA. Lifelines did not include an assessment of AHR, so only included a ClinR phenotype available for replication. The weighted Z-score method was used to meta-analyze the results of discovery and replication cohorts, considering EGEA used methylC-capture sequencing method, which was different from discovery and Lifelines (450 K array), to investigate DNA methylation. CpG sites that passed the epigenome-wide significance threshold of P < 1.14 × 10^–7^ (Bonferroni correction) in the meta-analysis of all studies were considered to be replicated. Finally, top sites identified in whole blood were verified in nasal brushes.

### Annotation and functional relevance

Significant CpG sites were annotated by GREAT v3.0.0 [18]. We correlated the significant CpG sites we identified to the expression level of gene nearby (with a region of ± 250 kb) by *cis* expression quantitative trait DNA methylation (*cis*-eQTM) analysis. Blood eQTM was assessed in the BIOS consortium dataset [19]. Matched nasal RNA sequencing data and DNA methylation data was used to perform eQTM analysis for significant nasal CpG sites and these results were additionally replicated in the larger collection of nasal brushes obtained in the PIAMA study [20]. The genes that were identified in eQTM analysis were used for pathway analysis by ConsensusPathDB [21]. To check if the results of replicated CpG sites were affected by inhaled corticosteroids (ICS), we stratified asthma subjects by ICS usage, and compared the DNA-methylation in the remission group to asthma with ICS and asthma without ICS group, respectively, by t-test. To evaluate the impact of BMI and allergic rhinitis on the results, we performed sensitivity analyses on the replicated blood CpG sites by additionally adjusting for allergic rhinitis and BMI respectively. We also performed sensitivity analysis for all significant nasal CpG sites.

## Results

### Subject characteristics

**Fig. 1:**
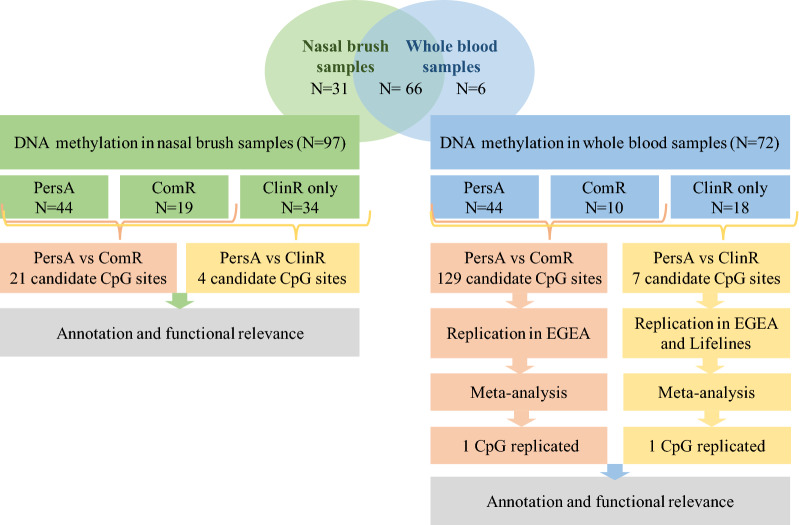
Study design. In the discovery panel,103 samples were assessed in this study, 72 subjects had whole blood DNA methylation data, 97 subjects had nasal DNA methylation data and 66 subjects have both. Epigenome-wide association studies were performed on clinical remission (ClinR) and complete remission (ComR) respectively in both whole blood and nasal samples. Significant CpG sites identified from whole blood were further replicated in two independent cohorts. Results of ComR were replicated in EGEA study (93 out of 129 probes available in EGEA); results of ClinR were replicated in EGEA study (4 out 7 probes available) and Lifelines (all 7 probes available)

Subject characteristics of the discovery and replication cohorts are shown in Table 1. Regarding the discovery cohort, the median [range] duration of follow-up of the entire population was 39 [4–49] years. Most of the subjects (group 1, n = 76) were from the study described by Carpaij et al. [4] at the third visit; the remaining (group 2, n = 27) were remission subjects added to this study to further enrich remission cases. Characteristics of subjects from the two groups are shown in Additional file 2: Table S1. By definition, group 2 had a higher proportion of remission subjects, and showed better lung function. Of the 103 subjects included in this study, 54 (52.4% of total subjects) had ClinR, and 20 of the ClinR subjects had ComR (19.4% of total subjects). At baseline, subjects in remission later had higher FEV_1_ and FVC level compared to PersA, but no difference regarding FEV1%predicted and FVC %predicted values was identified among the groups (Additional file 3: Figure S1). Within the whole dataset, 72 subjects (44 PersA, 28 ClinR and 10 of ClinR subjects are ComR) had whole blood methylation data and 97 (44 PersA, 53 ClinR and 19 of ClinR subjects are ComR) had nasal methylation data, with 66 subjects providing both (Fig. 1). White blood cell composition was estimated, and we did not observe significant cell composition differences among PersA, ClinR and ComR group in six cell types. (Additional file 3: Figure S2).

**Table 1:** Characteristics of study participants in the discovery and replication cohorts

	Discovery cohort			Lifelines			EGEA		
	PersA (n = 49)	ClinR (n = 54)	ComR (n = 20)	PersA (n = 99)	ClinR (n = 25)	Healthy (n = 636)	PersA (n = 106)	ClinR (n = 15)	ComR (n = 3)
Characteristics at last visit									
Age at remission status, years	49.8 (48.5; 51.3)	50.5 (48.3; 57.6)	50.5 (48.4; 59.8)	46.9 (40.7;50.7)	43.2 (35.7;50.3)	46.3 (39.2;52.9)	52.6 (36.7; 61.4)	49.2 (36.7; 57.6)	30.5 (28.2; 59.1)
Male, n (%)	31 (63.3%)	37 (68.5%)	15 (75.0%)	58 (58.6%)	10 (40.0%)	384 (60.4%)	49 (46.7%)	7 (46.7%)	1 (33.3%)
Duration of follow-up, years	39 (38;40.5)	39 (37; 42)	39 (37;41)				11.3 (10.7; 12.1)	11.6 (10.7; 12.2)	11.6 (10.0; 12.7)
Current-smoking, n (%)	4 (8.2%)	8 (14.8%)	2 (10.0%)	27 (27.3%)	14 (56.0%)	207 (32.5%)	10 (9.5%)	2 (13.3%)	1 (33.3%)
Ex-smoking, n (%)	13 (26.5%)	18 (33.3%)	7 (35.0%)	0 (0.0%)	0 (0.0%)	0 (0.0%)	35 (33.3%)	5 (33.3%)	0
Never-smoking, n (%)	32 (65.3%)	28 (51.9%)	11 (55.0%)	72 (72.7%)	11 (44.0%)	429 (67.5%)	60 (57.1%)	8 (53.3%)	2 (66.7%)
ICS, n (%)	31 (63.3%)	0	0	48 (48.5%)	0 (0.0%)	0 (0.0%)	72 (70.6%)	0	0
Allergic rhinitis, n (%)	33 (67.3%)	30 (55.6%)	9 (45.0%)	59 (59.6%)	13 (52.0%)	123 (19.3%)	70 (67.3%)	11 (73.3%)	2 (66.7%)
BMI	25.7 (23.7; 28.5)	26.8 (23.7; 28.3)	26.0 (22.8; 28.6)	25.7 (22.9;28.0)	26.0 (23.7;29.6)	25.8 (23.6;28.1)	23.9 (22.4; 27.5)	22.5 (19.9; 28.0)	22.0 (19.9; 22.4)
FEV1% pred	80.6 (71.1; 91.9)	*90.2 (82.9; 104.7)*	*101.8 (85.9; 110.0)*	81.2 (70.9;91.6)	87.1 (74.0;93.4)	101.5 (96.1;108.7)	87.0 (71.7; 98.4)	95.2 (87.5; 107.2)	97.5 (90.7; 121.5)
FVC% pred	94.2 (88.4; 103.7)	*101.7 (95.2; 111.5)*	*106.6 (101.2; 115.9)*	94.1 (85.8;102.2)	100.3 (90.4;109.2)	104.0 (97.5;111.4)	98.5 (84.8; 108.9)	95.2 (86.9; 106.1)	96.3 (86.9; 119.2)
FEV1/FVC	0.67 (0.60; 0.72)	*0.72 (0.68; 0.75)*	*0.72 (0.68; 0.76)*	0.67 (0.62;0.76)	0.67 (0.62;0.76)	0.79 (0.75;0.83)	0.69 (0.61; 0.77)	0.81 (0.78; 0.82)	0.80 (0.80; 0.91)
Characteristics at baseline									
Age, years	10.0 (9.0; 11.0)	11.0 (9.0; 17.5)	11.5 (9.8; 15.8)				40.6 (25.7; 48.7)	37.0 (26.0; 46.0)	18.9 (18.8; 46.4)
FEV1% pred	76.6 (67.4; 84.0)	77.1 (67.5; 83.8)	78.1 (73.5; 83.7)				81.7 (66.2; 95.9)	90.6 (82.0; 103.0)	82.0 (80.1; 122.9)
FVC% pred	88.8 (84.0; 96.1)	88,3 (80.9; 95.3)	88.9 (79.8; 96.5)				95.5 (83.7; 104.3)	86.4 (81.9; 100.6)	84.5 (70.1; 118.8)
FEV1/FVC	0.73 (0.64; 0.78)	0.73 (0.66; 0.79)	0.77(0.66; 0.80)				0.70 (0.63; 0.80)	0.86 (0.82; 0.89)	0.86 (0.83; 0.96)
Start asthma before age 16, n (%)	48 (98.0%)	50 (92.6%)	19 (95.0%)	59 (62.1%)	18 (81.8%)		52 (49.5%)	8 (53.3%)	2 (66.7%)

Continuous data are presented as median (25th percentile; 75th percentile), category data are presented as number (percentage)

Italic values represent significant differences compared with persistent asthma with P < 0.05 in the discovery cohort

### Differential methylation in the discovery cohort of ClinR

**Fig. 2:**
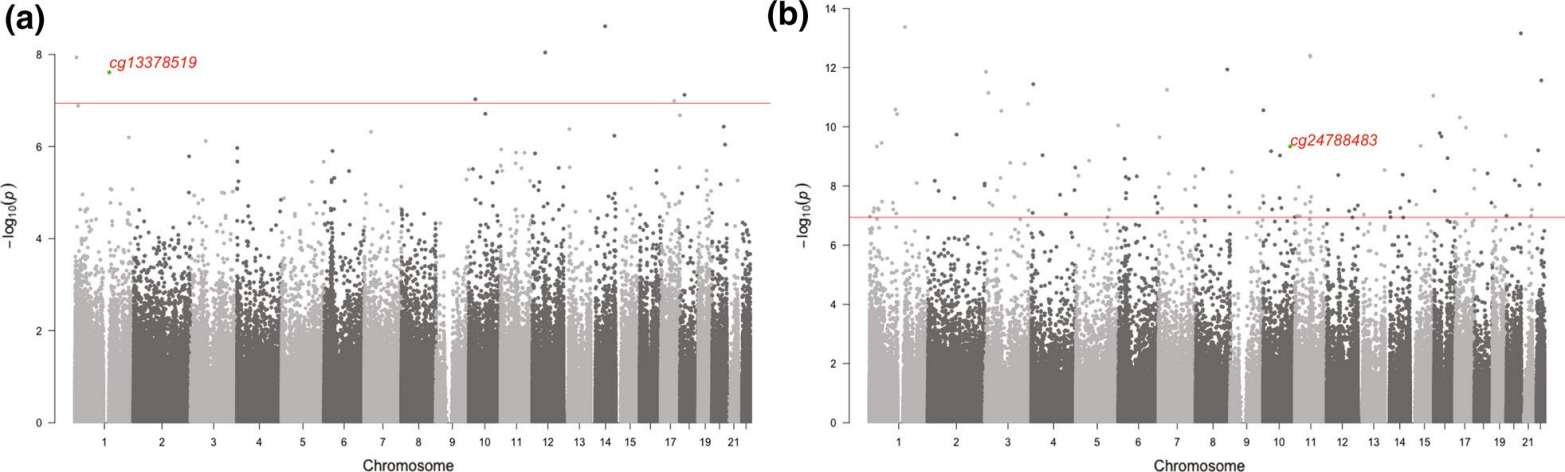
Manhattan plot of association between clinical remission (**a**)/ complete remission (**b**) and DNA methylation in whole blood in discovery cohort. In total, 436,824 CpGs were tested. The red line represents the genome-wide significance threshold (Bonferroni correction, P < 1.14 × 10^–7^). Highlighted sites represent replicate CpG sites

Whole blood DNA methylation levels at seven individual CpG sites and 19 differentially methylated regions (DMR) were significantly associated with ClinR (Fig. 2a, Table 2, Additional file 2: Table S2). The Q-Q plot (Additional file 3: Figure S3a) and the inflation factor λ of 1.064 indicated no obvious inflation of the results. Three out of the seven CpG sites were significantly correlated with gene-expression level in *cis* (FDR < 0.05), resulting in four CpG-gene pairs that showed negative correlation (Additional file 2: Table S3). These four eQTM genes were enriched in two pathways: G Protein Signaling Pathways and Hemostasis (P < 0.01, Additional file 2: Table S4).

**Table 2:** Seven genome-wide significant CpG sites (P value < 1.14 × 10^–7^) of ClinR in relation to methylation in whole blood

CpG	CHR	Great annotation (distance to TSS)	Discovery				Replication-EGEA				Replication-Lifelines				Meta-analysis	
			Coef	SE	P	N	Coef	SE	P	N^b^	Coef	SE	P	N	P.meta	Direction
cg10125195	12	*LACRT* (+ 133)	1.32E−02	2.30E−03	9.03E−09	72	1.098	0.748	0.142	80 (69/11)	2.73E−04	3.56E−03	0.939	124	1.58E−04	+ + +
cg12713893	18	*SNRPD1* (+ 44)	4.64E−03	8.63E−04	7.52E−08	72	7.127	5.359	0.184	120 (106/14)	4.19E−04	1.11E−03	0.705	124	2.90E−04	+ + +
cg15404785	14	*TRMT5* (− 279,717); *TMEM30B* (+ 20,765)	− 3.09E−02	5.17E−03	2.43E−09	72	0.423	0.150	0.005	105 (93/12)	− 4.93E−03	6.11E−03	0.420	124	7.58E−02	− + −
cg27252019	17	*MKS1* (+ 550)	− 3.86E−02	7.24E−03	1.01E−07	72	− 0.033	0.269	0.902	91 (80/11)	5.71E−03	1.01E−02	0.572	124	1.80E−02	– +
cg06185924	10	*ACBD5* (− 42)	1.10E−02	2.06E−03	9.36E−08	72	NA	NA	NA	106 (92/14)	− 4.72E−03	2.33E−03	0.043	124	1.05E−01	+ NA−
cg13378519^a^	1	*PEX11B* (+ 18)	− 5.99E−03	1.07E−03	2.43E−08	72	NA	NA	NA	119 (105/15)	− 1.08E−02	2.61E−03	3.57E-05	124	2.58E−11	−NA−
cg17900884	1	*ICMT* (− 91)	3.58E−03	6.28E−04	1.15E−08	72	NA	NA	NA	114 (101/13)	− 1.78E−04	9.86E−04	0.857	124	9.16E−04	+ NA−

P.meta: the P value of meta-analysis of results from discovery, EGEA and Lifelines by using weighted Z-score method

Direction: the direction of regression coefficient in discovery, EGEA and Lifelines

NA: the result for this probe was not available in EGEA

^a^The CpG site was replicated with meta P value < 1.14 × 10^–7^

^b^N (persA/ClinR), N varies from one CpG to another because of the methylC-capture sequencing method used and QC criteria in EGEA

We also identified four epigenome-wide significant CpG sites and 24 DMRs that associated with ClinR in nasal brushes (Table S5-S6). The Q-Q plot (Additional file 3: Figure S4a) and the λ of 1.125 indicated no obvious inflation of the results. We verified the CpG sites identified from whole blood samples in nasal brushes, and found that one CpG (cg15404785) associated with ClinR showed nominal significance (P < 0.05) and the same direction of effect in nasal brushes (Table S7). Four ClinR-associated CpG sites were correlated to expression level of four genes in *cis* (nominal significant P < 0.05) in nasal brushes, with one pair (cg07673230-*RPL30* (Ribosomal Protein L30)) also being nominal significant and in the same direction in the PIAMA dataset (Additional file 2: Table S8).

### Differential methylation in the discovery cohort of ComR

We identified 129 individual CpG sites and 53 regions that were significantly associated with ComR in whole blood DNA (Fig. 2b, Table 3, Additional file 2: Table S9, 10). Q-Q plot is shown in Additional file 3: Figure S3b and λ was 1.178. The eQTM analysis identified 99 CpG-gene pairs for 45 out of the 129 CpG sites (FDR < 0.05, Additional file 2: Table S3). These eQTM genes were enriched in eight pathways (P < 0.01, Additional file 2: Table S4), and the top pathways were Activation of SMO (Smoothen), Platelet Adhesion to exposed collagen, and Cilium Assembly.

**Table 3:** Top ten CpG sites in meta-analysis of ComR in relation to methylation in whole blood showing the same direction of effect in discovery and replication cohort

CpG	CHR	Great annotation (distance to TSS)	Discovery				Replication-EGEA				Meta-analysis	
			Coef	SE	P	N	Coef	SE	P	N^b^	P.meta	Direction
cg24788483^a^	10	*HABP2* (− 401132), *TCF7L2* (+ 201,644)	− 1.31E−02	2.11E−03	4.62E−10	54	− 1.348	0.544	0.013	56 (53/3)	8.49E−10	–
cg15223066	10	*ITGB1* (− 23,896), *NRP1* (+ 354472)	− 8.56E−03	1.39E−03	6.65E−10	54	− 0.658	0.470	0.161	92 (89/3)	1.13E−06	–
cg26909813	17	*RPRML* (-605)	− 2.68E−02	4.15E−03	1.06E−10	54	− 0.621	0.539	0.249	95 (92/3)	1.52E−06	–
cg02341571	15	*PATL2* (+ 150)	− 1.69E−02	2.71E−03	4.39E−10	54	− 0.253	0.388	0.515	64 (62/2)	2.59E−06	–
cg00269245	16	*SLC12A3* (− 41116), *NUP93* (+ 93986)	1.13E−02	1.86E−03	1.14E−09	54	0.832	1.109	0.453	70 (68/2)	4.62E−06	+ +
cg24833566	4	*MTNR1A* (− 152680), *FAT1* (+ 15608)	1.02E−02	1.71E−03	2.38E−09	54	0.658	0.758	0.386	77 (74/3)	6.87E−06	+ +
cg25881850	11	*KCNK4* (− 1426)	− 1.23E−02	2.23E−03	3.43E−08	54	− 0.838	0.816	0.304	68 (67/1)	9.09E−06	–
cg24730612	14	*FOS* (− 95503), *TMED10* (− 6640)	− 1.47E−02	2.50E−03	4.15E−09	54	− 0.260	0.489	0.595	62 (61/1)	1.09E−05	–
cg23250019	11	*SLC29A2* (+ 818)	2.15E−02	4.00E−03	7.62E−08	54	0.645	0.541	0.234	82 (79/3)	1.61E−05	+ +

P.meta: the P value of meta-analysis of results from discovery and EGEA by using weighted Z-scores method

Direction: the direction of regression coefficient in discovery and EGEA

A list of all 129 significant CpG sites identified in discovery cohort and the information of replication in EGEA is shown in Additional file 2: Table S9

^a^The CpG site was replicated with meta P value < 1.14 × 10^–7^

^b^N (persA/ComR), N varies from one CpG to another because of the methylC-capture sequencing method used and different QC criteria in EGEA

In nasal brushes, we identified 21 CpG sites and 62 DMRs that were associated with ComR (Additional file 2: Table S11, 12). Q-Q plot is shown in Additional file 3: Figure S4b and λ was 1.318. Eight of the ComR-associated CpG sites identified from whole blood also showed nominal significance in nasal brushes with the same direction of effect (Additional file 2: Table S13). Seven ComR-associated CpG sites were correlated to expression level of nine genes in *cis* (nominal significant P < 0.05) in nasal brushes, and four pairs also showed nominal significant and in the same direction in PIAMA dataset (Additional file 2: Table S8).

### Replication study

**Fig. 3:**
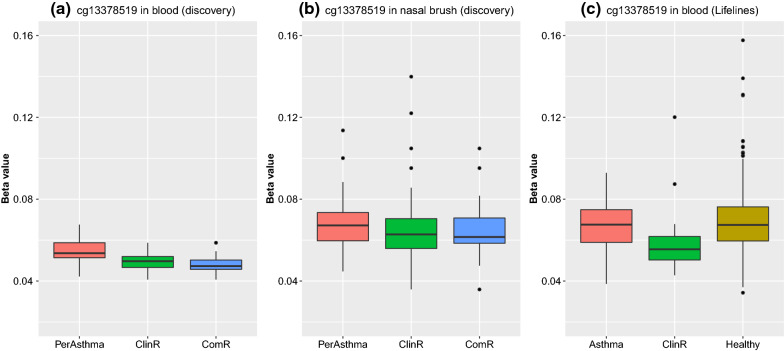
Boxplot illustrating DNA methylation levels of cg13378519 in persistent asthma, clinical remission and complete remission subjects. **a** DNA methylation levels in whole blood in discovery cohort; **b** DNA methylation levels in nasal brushes in discovery cohort; **c** DNA methylation levels in whole blood in replication cohort (Lifelines)

We replicated the top findings in whole blood in two independent cohorts. In replication cohort EGEA, four ClinR-associated CpG sites and 93 ComR-associated CpG sites were available, with one CpG site for ClinR and one for ComR showing nominal significance (P < 0.05) and the same direction as the discovery cohort. In the replication cohort Lifelines, which only had the ClinR phenotype, seven ClinR-associated CpG sites were all available with one CpG with P < 0.05 and the same direction. After meta-analysis, one CpG associated with ClinR and another one CpG associated with ComR were replicated (meta P < 1.14 × 10^–7^). The ClinR-associated CpG (cg13378519) was located on Chr 1 and close to gene *PEX11B* (Peroxisomal Biogenesis Factor 11 Beta). The CpG site was not available in the EGEA study and was only replicated in Lifelines. This CpG was lower methylated in ClinR subjects compared to PersA (Fig. 3a). A similar trend was also found in nasal brushes although the association was not statistically significant (Fig. 3b). The methylation levels at this CpG also differed between ClinR and healthy subjects in Lifelines cohort (Fig. 3c), indicating that DNA methylation status of asthma remission subjects is not equal to that of healthy subjects. The ComR-associated CpG (cg24788483) was located on chr10 and close to gene *HABP2* (Hyaluronan Binding Protein 2) and *TCF7L2* (Transcription Factor 7 Like 2). This CpG was lower methylated in ComR subjects compared to PersA in whole blood, but showed no obvious difference in nasal brushes (Additional file 3: Figure S5). In whole blood cis-eQTM analysis, this CpG was significantly negatively correlated to the expression level of *TCF7L2* gene (P = 9.12 × 10^–7^)*.*

To evaluate potential SNP effects within the probe for the two replicated CpG sites, the β value distributions were visually assessed in the discovery cohorts (Additional file 3: Figure S6), and no bimodal distribution was detected indicating no underlying SNP effect within the probe. The regional co-methylation plots of genes annotated to replicated CpG sites are shown in the Additional file 3: Figure S7, which showed that cg13378519 is located in the promoter region of *PEX11B* gene, and cg24788483 is located in heterochromatin region of *TCF7L2* gene. We did not identify any SNP that was associated with the two CpG sites using an online database [19].

We verified if the results of the two replicated CpG sites were affected by inhaled corticosteroids (ICS), known to affect DNA methylation at multiple sites in the genome (Additional file 3: Figure S8). The boxplots showed that the methylation levels in the remission groups (both ClinR and ComR) were significantly lower than both persistent asthma with ICS usage group and persistent asthma without ICS usage group, which indicated that the results were not affected by the use of ICS. We evaluated the impact of rhinitis on our results, as rhinitis shows comorbidity with asthma. The replicated ClinR-associated CpG site showed similar effect size and P value before and after adjusting for rhinitis, while the ComR-associated CpG showed similar effect size yet the P value changed from 4.62E-10 to 4.07E-05 after correction for rhinitis (Additional file 2: Table S14). Additional adjustment for BMI did not change the results of the two replicated CpG sites, indicating that BMI was not a confounder (Additional file 2: Table S14). The significant CpG sites identified from nasal samples showed similar effect size and P value before and after adjusting for BMI and for rhinitis (Additional file 2: Table S15).

We also compared our results from the discovery cohort with the published DNA methylation study of asthma remission in bronchial biopsies (Vermeulen et al. [10]). One CpG (cg13525448), located close to *LBX1* (Ladybird Homeobox 1) gene and *TLX1* (T Cell Leukemia Homeobox 1) gene, in their top list of remission vs persistent asthma reached nominal significance (P < 0.05) and showed the same direction of effect in our results of ComR in whole blood (Additional file 2: Table S16). Two genes of their DMRs associated with ClinR, at the gene *PTCHD3 (*patched domain-containing protein 3*)* and *LOC100507389*, were also present in our results of DMRs associated with ClinR in nasal brushes (Additional file 2: Table S6).

## Discussion

In this study, we identified one CpG site associated with ClinR and a different CpG site associated with ComR from whole blood, that were replicated in independent cohorts. We also revealed that 25 CpG sites were associated with asthma remission phenotypes in nasal brushes. In the nasal dataset, two differentially methylated regions were previously observed in another DNA methylation study of asthma remission using bronchial biopsies. This is the first epigenome-wide association study of asthma remission in both whole blood and nasal epithelial cells. This study is important since understanding asthma remission may provide new leads for future asthma treatments, and epigenetic studies may help to reveal these cellular mechanisms.

The two replicated CpG sites were annotated to genes which were not known to be involved in asthma (remission) before. ClinR-associated CpG cg13378519 was located in the promoter region of *PEX11B* gene. *PEX11B* encodes a protein of the PEX11 family [22]. Overexpression of the *PEX11B* gene in human cells induces peroxisome proliferation [23, 24]. Our results could indicate a possible role of peroxisomal proliferation as being involved in asthma remission. ComR-associated CpG cg24788483 was located in the *TCF7L2* gene and also correlated with the expression level of this gene. The *TCF7L2* gene product is a transcription factor that plays a role in the Wnt signaling pathway. SNPs in *TCF7L2* were previously reported to be strongly associated with type-2 diabetes and this gene plays a role in the pancreatic insulin secretory response to incretins [25, 26]. Previously, the risk of asthma was reported to be higher in type 2 diabetes patients [27] and it has been suggested that treatment targeting insulin resistance may have a positive effect on asthma patients [28]. However, it has never been studied whether this treatment could contribute to asthma remission. In addition, we could not infer from the current results of association analysis whether the DNA methylation signal we identified is the cause or consequence of asthma remission.

Although remission subjects do not have asthma symptoms any more, they still may be different from healthy people. Airway abnormalities such as basement membrane thickening still exist in clinical and complete remission subjects [29]. Vermeulen et al*.* [10] reported that the DNA methylation profile in remission subjects is different from that in healthy subjects. Our data in whole blood for our significantly replicated CpG confirmed this observation. The methylation levels of the ClinR associated CpG (cg13378519) were different between ClinR and healthy subjects, which indicated that the methylation status of subjects with asthma remission may not simply return to that of healthy people.

DNA methylation is cell-type specific. Both of the studied cells or tissues are highly heterogeneous and this may confound the association between DNA methylation and disease. Thus, we applied the widely used Houseman’s cell type correction method in blood samples, and for nasal brushes, we used SVA which showed good performance in cell type correction when reference data is lacking [30]. DNA methylation is also tissue specific. It is yet unknown if mechanisms of remission include local, tissue specific airway (epithelial) effects, immune cell effects, or both. Matched data from whole blood and nasal brush samples enable us to compare DNA methylation profiles associated with asthma remission in two different tissues, as proxies of both immunological and airway specific mechanisms. Although we identified several interesting CpG sites related to remission in nasal epithelium, our results of nasal brushes still need further replication. In previous studies, several DNA methylation sites were significantly associated with asthma in both whole blood and nasal cells [9, 31]. In our study, although no significant enriched cross-tissue effect of DNA methylation in asthma remission was shown, we did identify nine CpG sites in blood DNA that could be replicated in DNA of nasal brushed cells. This indicated that there may be shared DNA methylation signals in blood and nasal cells. We interpret these findings as either indicative of the presence of cross-tissue epigenetic mechanisms in blood and epithelial cells, or the possibility that the DNA methylation signal in nasal brushed cells is partly driven by immune cells in the nose [20], that are also present in blood.

DNA methylation might be related to the regulation of gene expression, and eQTM analysis may help to understand the function of CpG sites. Among the eQTM genes that correlated with ClinR-associated CpG sites in whole blood, the protein encoded by *PRKCH* (Protein Kinase C Eta) plays a key role in epithelial tight junction regulation which is important in maintaining the integrity and function of the airway epithelial barrier [32]; *PDE1B* (Phosphodiesterase 1B) encoded a protein belonging to phosphodiesterases (PDEs) family, and various PDE inhibitors showed anti-inflammatory, anti-remodelling and bronchodilator effect and are potential treatment of asthma [33]. In whole blood, 99 eQTM genes that were associated with ComR were enriched in eight pathways, among which three pathways were related to airway epithelial function: activation of SMO (Smoothen), cilium assembly and focal adhesion. The activation of SMO activity in bronchial epithelia enhanced the allergen-induced goblet cell metaplasia, which is defined as a reversible transformation of airway epithelial cells to mucous cells such as goblet cells and may occur in asthma [34]. Airway cilia are important for clearance of inhaled particles and pathogens. One study showed asthma patients had less ciliated cells in airway samples than healthy people [35], and changes in ciliary function may be relevant for the development of asthma in children [36]. Focal adhesion between the cell membrane and matrix are essential elements of airway smooth muscle cells migration which may play an important role during the airway remodeling of persistent asthma [37]. Blood is considered as an easily accessible surrogate tissue for asthma study, and previous studies showed some shared gene expression pattern and pathways between blood and airway/ lung [38, 39]. Our results suggest that genes related to asthma remission associated CpG sites may reflect a role for airway epithelial barrier and airway remodeling in asthma remission.

Corticosteroids treatment is usually used to control inflammation and improve control of asthma. One previous study identified differences in DNA methylation of CpG sites in blood associated with systemic corticosteroid treatment in patients with COPD, suggesting the potential effect of corticosteroids on the DNA methylation profile [40]. In our study, we therefore assessed the effect of ICS on the methylation levels of the two replicated CpG sites and found that the results were not affected by ICS usage. However, the effect of corticosteroid treatment on DNA methylation in nasal and blood samples of patients with asthma and remission is largely unknown and should be further investigated in the future.

Rhinitis and asthma often co-exist and show shared genetic origins [41]. A previous study of our group also showed a shared DNA methylation signal of asthma and rhinitis, suggesting the importance of considering the presence of rhinitis when doing EWAS of asthma in nasal epithelium [20]. In this study, we showed that additional adjustment for rhinitis changed the results on one of the replicated CpG sites (cg24788483) identified from blood. This remission associated CpG site was associated with active rhinitis in the absence of asthma, suggesting that blood DNA methylation profiles of persistent asthma and rhinitis overlap. This indicates that we should control for allergic rhinitis in future studies of asthma remission.

When comparing our results with those of Vermeulen et al. [10] in bronchial biopsies, one CpG (cg13525448) in their top list of remission vs persistent asthma reached nominal significance (P < 0.05) in our data and showed the same direction in our results of ComR in whole blood (Additional file 2: Table S16). This CpG site is annotated to *LBX1* gene and *TLX1* gene by position. Besides, among their DMRs associated with ClinR, regions at the gene *PTCHD3* and *LOC100507389* also showed up in our results of ClinR in nasal brushes. SNPs in *PTCHD3* gene were previously associated with asthma in African American children [42].

There are strengths and limitations in this study. To the best of our knowledge, this is the only cohort worldwide to study asthma remission in nasal brushes, which enable us to compare the DNA methylation profile in relation to asthma remission in whole blood and nasal epithelium. However, because of the uniqueness of this data, we could not replicate our results in nasal brushes in another independent cohort. Notably, we had matched DNA methylation and gene expression data in nasal brushes, which help us to get a better understanding of the function of CpG sites associated with asthma remission in nasal brushes. Regarding the limitations of this study, firstly, this study was performed in a relatively small sample with limited number of ComR cases, and the results were replicated in EGEA which is also with few ComR cases, which lead to low power of replication of ComR results. Secondly, although we studied over 450,000 DNA methylation sites, this only represents 1.6% of the human DNA methylome. Thirdly, we studied mixed contributions of cells in whole blood and nasal brushes; and although we corrected for cell types using established methods, potentially stronger results may be anticipated when studying pure cell types, as was previously shown for DNA methylation sites associated with asthma in purified blood eosinophils [8]. Finally, we used a cross sectional analysis at follow up to investigate asthma remission. Future studies, with prospective designs, should be performed to investigate if these DNA methylation sites can predict future asthma remission.

In conclusion, we identified replicable DNA methylation signals associated with clinical and complete asthma remission, which may play a role in peroxisome proliferation and Wnt signaling pathway. This could help in identifying the underlying mechanisms of asthma remission and also of the chronicity of the disease.

## Supplementary Information

**Additional file 1.** Supplementary materials.

**Additional file 2: Table S1. **Characteristics of subjects from two groups.** Table S2. **Differentially methylated regions associated with ClinR in whole blood identified by both comb-p (Sidak p-value) and DMRcate (FDR) methods.** Table S3 **Correlation analysis of DNA methylation and gene expression levels (in cis, +/- 250 kb) for asthma remission in blood.** Table S4**. Pathway analysis of eQTM genes correlated with ClinR and ComR in blood.** Table S5.** Genomewide significant (pvalue < 1.14E-07) CpGs associated with ClinR in nasal brushes.** Table S6.** Differentially methylated regions associated with ClinR in nasal brushes identified by both comb-p (Sidak p-value) and DMRcate (FDR) methods.** Table S7.** Look up of ClinR-assocaited CpGs (indentified from blood) in the results of nasal brushes.** Table S8**. Correlation analysis of DNA methylation and gene expression levels (in cis, +/- 250 kb) for asthma remission in nasal brushes.** Table S9.** 129 genome-wide significant (pvalue < 1.14E-07) ComR-associated CpGs in whole blood in discovery cohort.** Table S10.** Differentially methylated regions associated with ComR in whole blood identified by both comb-p (Sidak p-value) and DMRcate (FDR) methods.** Table S11.** Genome-wide significant (p value < 1.14E-07) CpGs associated with ComR in nasal brushes.** Table S12**. Differentially methylated regions associated with ComR in nasal brushes identified by both comb-p (Sidak p-value) and DMRcate (FDR) methods.** Table S13. **Look up of ComR-associated CpG sites (indentified from blood) in the results of nasal brushes.** Table S14.** Summary statistics of replicated CpGs (identified from blood) before and after adjusting for BMI and rhinitis.** Table S15.** Summary statistics of significant CpGs (identified from nasal samples) before and after adjusting for BMI and rhinitis.** Table S16.** Look up of CpGs associated with ClinR identified by Vermeulen et al. in this study.

**Additional file 3: Figure S1.** Lung function of different groups at baseline and last visit. In this figure, t-test was used in comparing means of any two groups (ns: P>0.05, *: P<0.05, **: P<0.01, ***: P<0.001, ****: P<0.0001).** Figure S2. **Estimated cell proportions among different groups. In this figure, t-test was used in comparing means of any two groups (ns: P>0.05).** Figure S3. **Quantile–quantile plot for epigenome-wide meta-analysis of the association between asthma remission and blood DNA methylation (n = 72). (a) ClinR, (b) ComR.** Figure S4.** Quantile–quantile plot for epigenome-wide meta-analysis of the association between asthma remission and nasal DNA methylation (n = 97). (a) ClinR, (b) ComR.** Figure S5.** Boxplot illustrating DNA methylation levels of cg24788483 in persistent asthma, clinical remission and complete remission subjects in discovery cohort. (a) DNA methylation levels in blood, (b) DNA methylation in nasal brushes. ** Figure S6. **Density distributions of DNA methylation levels of two replicated CpGs in 72 blood samples from discovery cohorts.** Figure S7. **Regional association plot of two replicated CpGs. For each plot from top to bottom the tracks included are: 1) Log10(P values) from the discovery 4 years model with CpGs indicated by dots. 2) Annotation tracks for the plotted genomic region taken from UCSC Genome Browser. 3) Pairwise correlation matrix across the displayed CpGs.** Figure S8. **DNA methylation levels of two replicated CpGs in remission subjects and asthma patients stratified by ICS usage. In this figure, t-test was used in comparing means of any two groups (*: P<0.05, ***: P<0.001, ****: P<0.0001).

## Data Availability

DNA methylation data has been deposited at the European Genome-phenome Archive (EGA), which is hosted by the EBI and the CRG, under accession number EGAS00001004766.
