# Supplementary material for: Epigenome-wide association study identifies DNA methylation markers for asthma remission in whole blood and nasal epithelium
Source: Clin Transl Allergy. 2020 Dec 11;10:60. doi: 10.1186/s13601-020-00365-4 (PMC7731549; doi:10.1186/s13601-020-00365-4)
Supplement: Supplementary file 1 — Additional file 1. Supplementary materials. [file 13601_2020_365_MOESM1_ESM.docx]

**Additional file 1: Supplementary materials**

**Supplementary Methods**

**Sample collection**

Nasal epithelial cells were collected by brushing. Briefly, the right nostril of the subjects was examined and the inferior turbinate was located using a speculum and penlight. Brushing was performed with Cytosoft brush CP-5B (Cyto-Pak) after local anesthesia with 1% lidocaine spray. The lateral area underneath the inferior turbinate was then brushed for 3 seconds and the brush was placed in a 2ml screw-cap Eppendorf tube and put into a freezer at -80°C until further processing. In total 4 brushes (2 for DNA isolation and 2 for RNA isolation) were collected.

**DNA methylation measurement and quality control**

DNA was extracted from 72 whole blood and 103 nasal brushing samples. DNA from whole blood was extracted using QIAamp blood kit (Qiagen, Benelux BV, Venlo, the Netherlands) and nasal epithelium samples was extracted using DNA investigator kit (Qiagen, Benelux BV, Venlo, the Netherlands). DNA concentration was determined by Nanodrop measurement and Picogreen quantification. 500 ng of DNA was bisulphite-converted using the EZ 96-DNA methylation kit (Zymo Research), following the manufacturer’s standard protocol. After verification of the bisulphite conversion step using Sanger Sequencing, Genome-wide DNA methylation was measured using the Illumina Infinium HumanMethylation450 beadchip (450K array; Illumina, Inc., San Diego, CA, USA). DNA concentration was normalized and the samples were randomized to avoid batch effects, and all paired (blood and epithelium) samples were hybridized on the same chip. One standard female DNA sample was included in this step for control reasons, to verify chip to chip variation.

DNA methylation data were pre-processed in R with the Bioconductor package Minfi^1^, using the original IDAT files extracted from the HiScanSQ scanner. We implemented sample filtering to remove bad quality samples (call rate <99%). Furthermore, we used both the 65 SNP probes to check for concordances between paired DNA brush and blood samples from the same individuals. Paired samples that showed a SNP signal with a Pearson correlation coefficient <0.9 were regarded as sample mix-ups and were excluded from the study. We also verified the methylation distribution of the X-chromosome to verify gender. We subsequently removed six samples due to call rate < 99%, none of samples were removed due to sample mix-up or gender mismatch. During processing, the low-quality probes (>10% of samples with detection p-value > 0.01; 1,842 probes), the probes on sex chromosomes (11,648 probes), the probes that mapped to multiple loci (29,233 probes), 65 SNP-probes and the probes containing SNPs at the target CpG sites with a MAF>5% (6,818 probes) were excluded^2^. We implemented “DASEN”^3^ to perform signal correction and normalization. After quality control, 72 blood samples and 97 nasal epithelium samples with 436,824 probes remained for following steps.

Methylation levels (beta values, β) at a given CpG site were derived from the ratio of the methylated probe intensity to overall fluorescence intensity (sum of methylated and unmethylated probe intensities): β is equal to M/ (U + M+ α), where M is intensity of the methylated probe, U is the intensity of the unmethylated probe, and α is the constant offset with the default value of 100. After association analysis, the inflation factor for each model was calculated using the function “estlambda” with method of “median” in R package GenABEL^4^.

**Nasal RNA sequencing and quality control**

Total RNA was extracted using AllPrep DNA/RNA Mini kit (Qiagen, Venlo, the Netherlands) from nasal brushing samples. Samples were lysed in 600 µl RLT-plus buffer using an IKA Ultra Turrax T10 Homogenizer, and RNA was purified according to the manufacturer’s instructions. RNA samples were dissolved in 30 µl RNAse free water. Concentrations and quality of RNA were checked using a Nanodrop ND-1000 and run on a Labchip GX (PerkinElmer, Waltham, MA).

Initial quality check of and RNA quantification of the samples was performed by capillary electrophoresis using the LabChip GX (PerkinElmer, Waltham, MA). Non-degraded RNA-samples with integrity scores RIN > 6 were selected for subsequent sequencing analysis. Sequence libraries were generated by Poly (A) enrichment using the TruSeq RNA Sample Prep Kit (Illumina, San Diego, CA) using the Sciclone NGS Liquid Handler (PerkinElmer, Waltham, MA). In case of contamination of adapter-duplexes, an extra purification of the libraries was performed with the automated agarose gel separation system Labchip XT (PerkinElmer, Waltham, MA). Whenever sample preparation failed, either a new sample preparation was attempted or the sample was replaced by a spare nose brush sample from the same individual. The obtained cDNA fragment libraries were loaded in pools of multiple samples unto Illumina HiSeq2500 sequencer using default parameters for paired-end sequencing (2 × 100 bp). If sequencing of a library gave insufficient reads, a second sequencing run was performed to generate additional reads, target was 15 million read pairs per sample.

The trimmed fastQ files where aligned to build b37 of the human reference genome using HISAT (version 0.1.5) allowing for 2 mismatches^4^. Reads from separate runs of the same library were merged. Before gene quantification, SAMtools (version 1.2) was used to sort the aligned reads^5^. The gene level quantification was performed by HTSeq (version 0.6.1p1) using –mode=union --stranded=no and using Ensembl version 75 as gene annotation database^6^.

Quality control (QC) metrics were calculated for the raw sequencing data, using the FastQC tool (version 0.11.3). Alignments of RNA of 86 subjects were obtained. QC metrics were calculated for the aligned reads using Picard-tools (version 1.130) (http://picard.sourceforge.net) CollectRnaSeqMetrics, MarkDuplicates, CollectInsertSize-Metrics and SAMtools flagstat. We discarded 17 samples due to poor alignment metrics. In addition, we checked for concordance between sex-linked (XIST and Y-chromosomal genes) gene expression and reported sex. Two more samples were discarded for lack of concordance. This resulted in high quality RNAseq data from 67 subjects.

Expressed features were excluded from analysis if less than half of the samples had counts per million mapped reads (CPM) of at least 5/M, where M is the median library size in millions. This left 17,479 expressed features for analysis. Raw count data were transformed to log2CPM using voom and analysed in the limma package^7^.

**Differentially methylated regions (DMR)**

Differentially methylated regions (DMR) were identified using comb-p v0.48^8^ and DMRcate^9^. For comb-p, we set the seed P value as 0.05, the adjusted region-level P value cutoff to determine DMR (Sidak-corrected P) as 0.05 and the maximum distance to search for adjacent peaks as 1000 bp. For DMRcate, we set the width of estimation as 1000 bp, and P value cutoff to determine DMR as 0.01. Comb-p and DMRcate use different algorithms to identify significantly DMRs. Comb-p uses a one-step Šidák correction method for multiple comparisons^10^, while DMRcate uses FDR method. To reduce false positives, we only considered a DMR to be statistically significant if it was significant by both methods, according to the definition used in each^11^.

**Replication and meta-analysis**

Genome-wide significant CpG sites that passed Bonferroni correction (P< 1.14×10^-7^, which is 0.05 / 436,824) in whole blood DNA were selected for replication in two cohorts Lifelines and EGEA (see below for cohort description). We replicated results of ClinR in both cohorts and replicated results of ComR only in EGEA cohort since Lifelines did not have a ComR phenotype. The meta-analysis was performed using weighted Z-score method. For results of ClinR, results of three CpG probes were not available in EGEA cohort, and we only meta-analyze the results of these three CpG sites in discovery and Lifelines.

**Cohort description**

**1) Epidemiological study on the Genetics and Environment of Asthma (EGEA) cohort**

*Population*

The Epidemiological Study on the Genetics and Environment of Asthma (EGEA: <https://egeanet.vjf.inserm.fr> ) is a French 20-year follow-up cohort that included at baseline (early 1990s) a group of asthmatic patients with their first-degree relatives and a group of control subjects. 2047 adults and children were recruited from 1991 to 1995 (EGEA1). A first follow-up of the EGEA population was conducted from 2003 to 2007 (EGEA2; 1845 subjects), and a second follow-up was conducted from 2011 to 2013 (EGEA3; 1558 subjects). At each survey a detailed respiratory questionnaire (self-completed in EGEA3) was included, and in the 2 first surveys a clinical examination including lung function testing, measure of bronchial responsiveness, skin prick tests, and total IgE measurement was performed. No follow-up bias related to asthma status and asthma-related phenotypes was observed. A rich biobank, including blood samples, has been constituted (BB-0033-00043). The EGEA study was approved by the appropriate ethics committees. The current analysis was conducted among asthma participants with available data to define asthma remission at EGEA2, and with available methylome data (n=124, including 106, 15 and 3 individuals with PersA, ClinR and CompR).

*Phenotype definition*

**Asthma:** subjects who used asthma medications in the past 12 months or who had asthma symptoms (asthma attacks or wheezing in the chest) in the past 12 months.

**Clinical remission:** subjects who did not use asthma medication and did not have asthma symptoms (asthma attacks or wheeze).

**Complete remission:** no asthma medication, no asthma symptoms (asthma attacks or wheeze), no BHR and FEV1 % predict >80% (GLI 2012).

*Methylome data*

The DNA was extracted from buffy coat and high-resolution methylome data in regulatory elements of immune cells was generated by MethylC-capture sequencing (MCC-Seq)^12^. Overall, the panel designed covered 4,861,805 CpGs.

Regarding quality control, DNA methylation of each CpG was measured by the number of methylated reads over the total number of sequenced reads. We filtered CpGs with the number of total sequenced reads are less than 5X. We further filtered the CpGs if the difference of methylation level between forward strand and reverse strand is great than 20% for CpGs with >=2X of both forward strand and reverse strand. In addition, CpGs overlapping SNPs from dbSNPs (137) with minor allele frequency (MAF) >0.01 and CpGs located within ENCODE DAC blacklisted regions or Duke excluded regions were discarded.

*Statistical analysis*

For each CpG site, we fitted a generalized linear model assuming a binomial distribution of methylation levels (considering the proportion of methylated reads weighted for sequence read coverage as the dependent variable), and asthma remission outcome as independent variable. Models were adjusted for age, sex, smoking status (never smokers versus ever smokers) and measured blood proportions of monocytes, lymphocytes, neutrophils, eosinophils and basophils. The R^13^ function glmer in the lm4 package^14^ was used. The R code for the association was: glmer (cbind (Methylated_Read_Counts, Unmethylated_Read_Counts) ~ Asthma_Remission_Status + age + sex + smoking + neutro + lympho + mono + eosino + baso). The convergence of the model was performed by the BOBYQA algorithm^15^ which was found the most efficient on our data in terms of convergence errors and warnings. All continuous variables used in the model were individually centered (subtracting the mean of the variable) and scaled (dividing by the standard deviation of the variable) in order to increase the stability of the model and to decrease the computational time. P-values were calculated using the Wald test.

**2) Asthma remission methylation project (Lifelines)**

*Population and measurements*

In total, 1,656 subjects were selected of the Dutch population-based cohort study Lifelines^16^. All subjects provided written informed consent and the study was approved by the Medical Ethics Committee of the University Medical Center Groningen, Groningen, the Netherlands. Subjects were selected from the larger cohort based on having complete data on sex, age, height, smoking history (pack-years, never- or current-smoker), airway obstruction (FEV_1_/FVC<70%), and occupational related exposures. Self-reported never-smokers with 0 pack-years of smoking and current-smokers with a smoking history greater than 5 pack-years were selected. Ex-smokers were not included.

*Genome-wide methylation assay*

The Illumina Infinium Human Methylation 450K arrays were used to define genome-wide DNA methylation levels at >450.000 specific CpG sites. We randomized 1,656 subjects based on sex, exposure, and airway obstruction over the chips. Using 500 ng DNA for each sample, we first performed a bisulphite conversion using the EZ- 96 DNA methylation kit (Zymo research Corporation, Irvine, USA), which was validated using commercially available bisulphite conversion 1 control samples (Zymo Research Corporation, Irvine, USA). After this step the samples were processed according to the Illumina 450K protocol (Illumina Inc., San Diago, USA). Quality-control (QC) steps were performed using the Minfi package in R^1^, and included the removal of samples with >1% of all probes having a detection p-value >0.01, and samples with an incorrect sex or SNP prediction. We removed single probes with a detection p-value >0.01, sex chromosome probes, cross-reactive probes, probes measuring SNPs, and probes where the CpG itself or the single base extension (SBE) site is a SNP^2^. The data were normalized using DASEN implemented in the wateRmelon package in R^3^. The final data set contained data for 1,561 subjects and 420,938 CpG probes. Beta-values were used to represent DNA methylation levels, which is the ratio between the intensities of methylated versus unmethylated probes, ranging from 0 to 1.

*Phenotype definition*

**Healthy:** 1) no doctor’s diagnosed asthma, 2) no symptoms, 3) no use of asthma medication, 4) FEV1%pred > 90% (based on GLI-2012), 5) FEV1/FVC > lower limit of normal (based on GLI-2012). Based on this definition we have in total 636 healthy subjects in Lifelines.

**Active asthma:** doctor’s diagnosed asthma AND (2 or more symptoms OR use of asthma medication). Based on this definition we have in total 99 subjects with active asthma in Lifelines, of whom 48 use inhaled corticosteroids (combination beta-agonists/inhaled corticosteroids OR inhaled corticosteroids). Symptoms included: wheeze (Did you have this wheezing while you did not have a cold? Have you ever suffered from wheezing on the chest?), shortness of breath at rest (Have you ever had an attack of shortness of breath at rest during the day?), and woken by an attack (Have you ever woken up with an attack of shortness of breath?).

**Clinical Remission:** doctor’s diagnosed asthma AND no symptoms AND no use of asthma medication. Based on this definition we have in total 25 subjects with clinical remission in Lifelines.

*Statistical analysis*

Robust linear regression model was used to determine differential methylation between asthma and clinical remission. Model was adjusted for age, gender, smoking status, pack years, first 7 PC of methylation data and the percentage of monocytes, B cells, NK cells, CD4+ T cells, CD8+ T cells, neutrophils and eosinophils, which were predicted by the Houseman^17^ algorithm.

**Functional annotation and pathway analysis**

*Annotation by position*

Significant CpG sites were firstly annotated by GREAT 3.0.0^18^. Top sites identified in whole blood were further looked up in nasal brushes. Regional association plots of replicated CpG cites were generated using R package coMET^19^. In the association plot we included 15 CpG sites upstream to the target CpG site and 15 CpG sites downstream to the target CpG site. The association of replicated CpG sites identified from whole blood with SNP nearby (+/- 250kb) were checked in BIOS qtl browser (<https://genenetwork.nl/biosqtlbrowser/>), which is called *cis*-MeQTL^20^.

*cis-eQTM analysis*

After annotating each significant CpG to its nearest genes, we also annotated the CpG by the expression of nearby genes, which is called *cis* expression quantitative trait methylation (*cis*-eQTM). For blood *cis*-eQTM, we used DNA methylation and gene expression data from 3,075 samples in the Biobank-based integrative omics study (BIOS) consortium from the following cohorts: Leiden Longevity Study, Lifelines Study, Rotterdam Study, and Netherland Twin Study^20^. *cis*-eQTMs for significant CpG sites identified in blood were conducted for gene expression transcripts within ± 250 kb of each CpG site. In each cohort, the methylation M value (log2 ratio of methylated versus unmethylated probe intensities) was regressed on gene expression and adjusted for age, sex, lymphocyte proportion, monocyte proportion, and RNA flow cell number. The results from each cohort were meta-analyzed using an inverse variance-weighted fixed-effects model in METAL^21^. We accounted for multiple testing by controlling for the false discovery rate (FDR) at 0.05.

For nasal brushes we have matched DNA methylation and RNAseq data in the discovery cohort. We then assessed the association of DNA methylation with gene expression *cis* (+/-250kb from the CpG) for all significant CpGs identified in nasal EWAS. In total 63 samples had matched nasal DNA methylation and nasal RNAseq data. We performed linear regression analysis, and the model is: gene expression level ~ DNA methylation M values + age + gender + smoking + pack years. Because of the small sample size in discovery cohort, we select the CpG-gene pairs at a nominal significant level (P<0.05), and then replicated these pairs in PIAMA dataset^22^ which consists of 244 samples with matched DNA methylation and gene expression data.

The eQTM genes were used for pathway analysis by ConsensusPathDB^23^.

1. Aryee MJ, Jaffe AE, Corrada-Bravo H, et al. Minfi: a flexible and comprehensive Bioconductor package for the analysis of Infinium DNA methylation microarrays. *Bioinformatics*. 2014;30(10):1363-1369. doi:10.1093/bioinformatics/btu049

2. Chen Y, Lemire M, Choufani S, et al. Discovery of cross-reactive probes and polymorphic CpGs in the Illumina Infinium HumanMethylation450 microarray. *Epigenetics*. 2013;8(2):203-209. doi:10.4161/epi.23470

3. Pidsley R, Y Wong CC, Volta M, Lunnon K, Mill J, Schalkwyk LC. A data-driven approach to preprocessing Illumina 450K methylation array data. *BMC Genomics*. 2013;14(1):293. doi:10.1186/1471-2164-14-293

4. Aulchenko YS, Ripke S, Isaacs A, van Duijn CM. GenABEL: an R library for genome-wide association analysis. *Bioinformatics*. 2007;23(10):1294-1296. doi:10.1093/bioinformatics/btm108

5. Kim D, Langmead B, Salzberg SL. HISAT: a fast spliced aligner with low memory requirements. *Nat Methods*. 2015;12(4):357-360. doi:10.1038/nmeth.3317

6. Li H, Handsaker B, Wysoker A, et al. The Sequence Alignment/Map format and SAMtools. *Bioinformatics*. 2009;25(16):2078-2079. doi:10.1093/bioinformatics/btp352

7. Anders S, Pyl PT, Huber W. HTSeq--a Python framework to work with high-throughput sequencing data. *Bioinformatics*. 2015;31(2):166-169. doi:10.1093/bioinformatics/btu638

8. Ritchie ME, Phipson B, Wu D, et al. limma powers differential expression analyses for RNA-sequencing and microarray studies. *Nucleic Acids Res*. 2015;43(7):e47. doi:10.1093/nar/gkv007

9. Pedersen BS, Schwartz DA, Yang IV, Kechris KJ. Comb-p: software for combining, analyzing, grouping and correcting spatially correlated P-values. *Bioinformatics*. 2012;28(22):2986-2988. doi:10.1093/bioinformatics/bts545

10. Peters TJ, Buckley MJ, Statham AL, et al. De novo identification of differentially methylated regions in the human genome. *Epigenetics Chromatin*. 2015;8:6. doi:10.1186/1756-8935-8-6

11. Šidák Z. Rectangular Confidence Regions for the Means of Multivariate Normal Distributions. *Journal of the American Statistical Association*. 1967;62(318):626-633. doi:10.1080/01621459.1967.10482935

12. Reese SE, Xu C-J, den Dekker HT, et al. Epigenome-wide Meta-analysis of DNA Methylation and Childhood Asthma. *Journal of Allergy and Clinical Immunology*. Published online December 2018. doi:10.1016/j.jaci.2018.11.043

13. Allum F, Shao X, Guénard F, et al. Characterization of functional methylomes by next-generation capture sequencing identifies novel disease-associated variants. *Nat Commun*. 2015;6:7211. doi:10.1038/ncomms8211

14. R Core Team. *R: A Language and Environment for Statistical Computing*. R Foundation for Statistical Computing; 2017. https://www.R-project.org/

15. Bates D, Mächler M, Bolker B, Walker S. Fitting Linear Mixed-Effects Models Using **lme4**. *J Stat Soft*. 2015;67(1). doi:10.18637/jss.v067.i01

16. Powell MJ. The BOBYQA algorithm for bound constrained optimization without derivatives. *Cambridge NA Report NA2009/06, University of Cambridge, Cambridge*. Published online 2009.

17. Stolk RP, Rosmalen JGM, Postma DS, et al. Universal risk factors for multifactorial diseases: LifeLines: a three-generation population-based study. *European Journal of Epidemiology*. 2008;23(1):67-74. doi:10.1007/s10654-007-9204-4

18. Houseman E, Accomando WP, Koestler DC, et al. DNA methylation arrays as surrogate measures of cell mixture distribution. *BMC Bioinformatics*. 2012;13(1):86. doi:10.1186/1471-2105-13-86

19. McLean CY, Bristor D, Hiller M, et al. GREAT improves functional interpretation of cis-regulatory regions. *Nature Biotechnology*. 2010;28(5):495-501. doi:10.1038/nbt.1630

20. Martin TC, Yet I, Tsai P-C, Bell JT. coMET: visualisation of regional epigenome-wide association scan results and DNA co-methylation patterns. *BMC Bioinformatics*. 2015;16(1):131. doi:10.1186/s12859-015-0568-2

21. the BIOS Consortium, Bonder MJ, Luijk R, et al. Disease variants alter transcription factor levels and methylation of their binding sites. *Nat Genet*. 2017;49(1):131-138. doi:10.1038/ng.3721

22. Willer CJ, Li Y, Abecasis GR. METAL: fast and efficient meta-analysis of genomewide association scans. *Bioinformatics*. 2010;26(17):2190-2191. doi:10.1093/bioinformatics/btq340

23. Qi C, Jiang Y, Yang IV, et al. Nasal DNA methylation profiling of asthma and rhinitis. *J Allergy Clin Immunol*. Published online January 14, 2020. doi:10.1016/j.jaci.2019.12.911

24. Herwig R, Hardt C, Lienhard M, Kamburov A. Analyzing and interpreting genome data at the network level with ConsensusPathDB. *Nature Protocols*. 2016;11(10):1889-1907. doi:10.1038/nprot.2016.117
