# Supplementary material for: Epigenome-wide association study identifies DNA methylation markers for asthma remission in whole blood and nasal epithelium
Source: Clin Transl Allergy. 2020 Dec 11;10:60. doi: 10.1186/s13601-020-00365-4 (PMC7731549; doi:10.1186/s13601-020-00365-4)
Supplement: Supplementary file 3 — Additional file 3: Figure S1. Lung function of different groups at baseline and last visit. In this figure, t-test was used in comparing means of any two groups (ns: P>0.05, *: P<0.05, **: P<0.01, ***: P<0.001, ****: P<0.0001). Figure S2. Estimated cell proportions among different groups. In this figure, t-test was used in comparing means of any two groups (ns: P>0.05). Figure S3. Quantile–quantile plot for epigenome-wide meta-analysis of the association between asthma remission and blood DNA methylation (n = 72). (a) ClinR, (b) ComR. Figure S4. Quantile–quantile plot for epigenome-wide meta-analysis of the association between asthma remission and nasal DNA methylation (n = 97). (a) ClinR, (b) ComR. Figure S5. Boxplot illustrating DNA methylation levels of cg24788483 in persistent asthma, clinical remission and complete remission subjects in discovery cohort. (a) DNA methylation levels in blood, (b) DNA methylation in nasal brushes. Figure S6. Density distributions of DNA methylation levels of two replicated CpGs in 72 blood samples from discovery cohorts. Figure S7. Regional association plot of two replicated CpGs. For each plot from top to bottom the tracks included are: 1) Log10(P values) from the discovery 4 years model with CpGs indicated by dots. 2) Annotation tracks for the plotted genomic region taken from UCSC Genome Browser. 3) Pairwise correlation matrix across the displayed CpGs. Figure S8. DNA methylation levels of two replicated CpGs in remission subjects and asthma patients stratified by ICS usage. In this figure, t-test was used in comparing means of any two groups (*: P<0.05, ***: P<0.001, ****: P<0.0001). [file 13601_2020_365_MOESM3_ESM.docx]

**Additional file 3:**

**Figure S1. Lung function of different groups at baseline and last visit.** In this figure, t-test was used in comparing means of any two groups (ns: P>0.05, *: P<0.05, **: P<0.01, ***: P<0.001, ****: P<0.0001)

**Figure S2. Estimated cell proportions among different groups.** In this figure, t-test was used in comparing means of any two groups (ns: P>0.05)

**Figure S3. Quantile–quantile plot for epigenome-wide meta-analysis of the association between asthma remission and blood DNA methylation (n = 72).** (a) ClinR, (b) ComR.

**Figure S4. Quantile–quantile plot for epigenome-wide meta-analysis of the association between asthma remission and nasal DNA methylation (n = 97).** (a) ClinR, (b) ComR.

**Figure S5. Boxplot illustrating DNA methylation levels of cg24788483 in persistent asthma, clinical remission and complete remission subjects in discovery cohort.** (a) DNA methylation levels in blood, (b) DNA methylation in nasal brushes.

**Figure S6. Density distributions of DNA methylation levels of two replicated CpGs in 72 blood samples from discovery cohorts.**


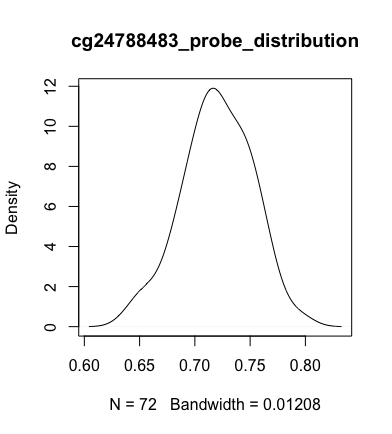

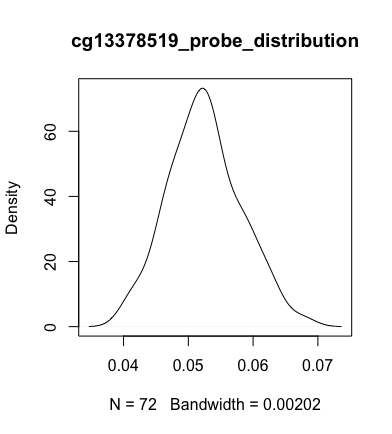


**Figure S7. Regional association plot of two replicated CpGs.** For each plot from top to bottom the tracks included are: 1) Log10(P values) from the discovery 4 years model with CpGs indicated by dots. 2) Annotation tracks for the plotted genomic region taken from UCSC Genome Browser. 3) Pairwise correlation matrix across the displayed CpGs.

**Figure S8. DNA methylation levels of two replicated CpGs in remission subjects and asthma patients stratified by ICS usage.** In this figure, t-test was used in comparing means of any two groups (*: P<0.05, ***: P<0.001, ****: P<0.0001)
